# The nuclear receptor NR6A1 plays an oncogenic role through reprogramming glycolysis in tumors

**DOI:** 10.1186/s40659-025-00646-x

**Published:** 2025-11-11

**Authors:** Xiaowen Liu, Ye Li, Xinxu Rao, Shun Xie, Zhuoxian Rong, Lifang Yang, Zenghui enghui Mao, Dan Li

**Affiliations:** 1 Hunan Provincial Key Laboratory of Regional Hereditary Birth Defects Prevention and Control, Changsha Hospital for Maternal & Child Health Care Affiliated to Hunan Normal University, Changsha, 410007 China; 2 Department of Life Science, College of Biology, Hunan University, Changsha, 410082 China; 3 Hunan Research Center of the Basic Discipline for Cell Signaling, Hunan University, Changsha, China; 4 Key Laboratory of Molecular Radiation Oncology, (Xiangya Hospital, Central South University), Changsha, 410082 China; 5 Cancer Research Institute, Xiangya School of Basic Medical Sciences, Central South University, Changsha, 410078 China

**Keywords:** NR6A1, mTOR, miR-302a, HK1, Glycolysis, Oncogenic role

## Abstract

NR6A1 is a member of the nuclear receptor superfamily of ligand-activated transcription factors and can bind to conserved DNA sequences, acting as a transcriptional repressor. NR6A1 has been reported to promote prostate cancer, gastric cancer, and testicular germ cell tumor progression. In the present study, we confirmed the oncogenic role of NR6A1 in several types of cancer cells, including HeLa, TFK1, and A549 cells, and revealed that NR6A1 knockdown led to a decrease in lung adenocarcinoma cell proliferation, a reduction in glucose consumption, a reduction in lactic acid production, and decreases in ATP levels and mitochondrial membrane potential. Mechanistically, through a series of methods, including bioinformatics analysis, dual-luciferase reporter gene assay, RT-qPCR, Western blot analysis, and functional rescue experiments, we demonstrated that NR6A1 may promote the expression of HK1 by directly suppressing miR-302a, thereby reprogramming tumor cell glycolysis and enhancing lung adenocarcinoma cell growth. In addition, we found that NR6A1 can affect mTOR signaling, suggesting a broader role in tumor metabolism regulation. In summary, our data indicate that NR6A1 plays an oncogenic role by reprogramming glycolysis via the miR-302a/HK1 axis in lung adenocarcinoma.

## Introduction

Metabolic changes are a common feature of most malignant tumors [1]. The Warburg effect, which contributes to the development and progression of tumors, is a metabolic phenomenon in which glycolysis still predominates even under normoxia [2, 3]. An increasing number of studies suggest that a variety of transcription factors, such as HIF-1α, p53, and SIX1, are highly expressed in tumors and are closely related to the enhancement of glycolysis through the regulation of many metabolic enzymes, mitochondrial production and function, and oxygen consumption [4–6].

Nuclear receptor subfamily 6 group member 1 (NR6A1), also termed germ cell nuclear factor (GCNF), is a ligand-dependent transcription factor [7, 8]. During embryonic development, NR6A1 is required for the maintenance of somitogenesis and subsequent development [9]. Loss of NR6A1 in mice leads to defects in the closure of neural tubes and embryonic lethality [10]. In adult mammals, NR6A1 expression is predominantly found in germ cells, and mutations in NR6A1 in oocytes cause a decline in female fertility [11, 12]. In 2013, Mathieu R et al. identified NR6A1 as a new member of the cancer/testis (CT) gene family in prostate cancer [13]. Subsequently, Cheng G et al. investigated the expression of NR6A1 and its function in prostate cancer (PCa) patients who underwent radical prostatectomy. They reported that NR6A1 expression was significantly associated with the Gleason score (GS) and tumor stage. Moreover, NR6A1 plays a prominent role in the epithelial‒mesenchymal transition (EMT), migration, and invasion of PCa cells [14]. During the development of gastric cancer (GC), the cross-talk between hsa_circ_001653 and NR6A1 was assessed, and the overexpression of NR6A1 restored the proliferation, migration, and invasion of GC cells lacking hsa_circ_001653 [15]. Previous studies in our laboratory have shown that NR6A1 promotes the progression of testicular germ cell tumors (TGCTs) by targeting E-cadherin [16]. These studies suggest that NR6A1 is closely related to the occurrence and development of tumors. Nuclear receptors affect the progression of tumor cells by participating in tumor glucose metabolism. For example, in pancreatic cancer, inhibiting the expression of NR3C2 leads to the upregulation of glycolytic enzymes HK1, HK2, and LDHA [17], promotes glucose uptake and lactate production and accelerates tumor growth. In addition, members of the nuclear receptor family, such as SRC-3 and orphan receptors, and their co-regulatory factors (NCOA) have become key targets for reversing the reprogramming of tumor glucose metabolism by directly regulating glycolytic enzymes or intervening in metabolism-related pathways (such as MAPK-ERK and p53). Targeting NR3C2 can effectively inhibit glycolysis hyperactivity [18], while combined intervention with multiple targets (such as PFKFB4 and NR2E3) may enhance therapeutic efficacy. This indicates that studying the relationship between nuclear receptors and tumor cell glycolysis is an effective strategy for exploring tumor targets and their occurrence and development. In liver cancer, NR6A1 has been reported to be a novel regulator of lipogenesis in HepG2 cells, and NR6A1 knockdown can increase lipid accumulation as well as insulin-induced proliferation and migration of HepG2 cells [19]. However, the role of NR6A1 in tumor glucose metabolism is poorly defined and requires additional research.

Cell metabolic reprogramming is a pivotal marker of malignant tumors [20]. The Warburg effect, a metabolic feature of cancer cells, consumes substantial amounts of glucose and produces lactate under aerobic conditions to meet increasing demands for energy and biosynthetic precursors [21]. The potential prognostic value of mTOR, a protein kinase, and its activated form phosphorylated mTOR (p-mTOR) has been extensively studied across various cancers, including lung cancer [22], other cancer types [23–27], and urological cancers [28].The mTOR signaling pathway is closely associated with growth factors, nutrients, and energy availability governing cell survival, growth, proliferation, and death. Previous clinical studies revealed that upregulation of the PI3K/Akt-mTOR pathway is prevalent in NSCLC patients. This pathway regulates diverse cellular physiological processes by activating downstream effectors, playing a critical role in tumor proliferation, invasion, and metastasis. Stimulation of class I PI3K activates its downstream effector Akt, subsequently leading to phosphorylation of mTOR at Ser2448—a crucial event in mTOR activation (p-mTOR) [29, 30]. Consequently, the mTOR pathway further regulates physiological and pathological events through activation by various exogenous stimuli and essential signaling pathways. Elevated mTOR/p-mTOR expression is increasingly recognized in association with specific cancers [1]. Aberrant mTOR pathway activation, induced by loss of tumor suppressors or oncogenic stimulation, significantly promotes tumor growth, angiogenesis, and metastasis [31]. Researchers have revealed that cancer cells exploit the Warburg effect to sustain PI3K signaling pathway activity, thereby promoting glycolytic metabolism to ensure continuous growth and division [32, 33]. mTORC1 activation elevates hypoxia-inducible factor-1α (HIF-1α) levels, inducing aerobic glycolysis in tumor cells [34]. Activated mTORC1 also regulates the expression levels of glycolysis-related enzymes in tumor cells through downstream transcription factor regulation. These findings underscore the instrumental role of PI3K/Akt-mTOR in metabolic regulation. Accordingly, targeting the PI3K/Akt-mTOR pathway to modulate tumor metabolism has emerged as a therapeutic strategy for cancers. Dysregulation of the mTOR signaling pathway occurs across various malignancies and correlates with poor patient prognosis. Therefore, investigating the regulatory mechanisms of the mTOR signaling pathway in tumor glycolysis is essential.

In the present study, we have investigated the function of NR6A1 in several types of cancer cells, including cervical cancer, Cholangiocarcinoma cells, and lung cancer cells, to confirm whether NR6A1 is upregulated in various cancer cells. The relationships between NR6A1 and tumor glycolysis, as well as the regulatory mechanism in lung cancer cells, were subsequently investigated. We demonstrated that NR6A1 plays an oncogenic role, that it activates p-mTOR, and increases the levels of HK1 in these cancer cells.

## Materials and methods

### Cell culture and treatment

The human cervical cancer line HeLa (ATCC^®^ CRM-CCL-2) and the intrahepatic bile duct cancer cell line TFK1 (ATCC^®^ CRL-5844) were cultured in Dulbecco’s modified Eagle’s medium (DMEM, HyClone, USA) supplemented with 10% heat-inactivated fetal bovine serum (Gibco, USA). The lung adenocarcinoma cell line A549 (ATCC^®^ CCL-185) was cultured in RPMI 1640 (HyClone, USA) supplemented with 10% heat-inactivated fetal bovine serum (Gibco, USA). All the cells were cultured at 37 °C in a 5% CO_2_ incubator (Thermo, USA). All the cell lines used were from the Cancer Research Institute, Xiangya School of Medicine, Central South University (Changsha, China).

For siRNA, miRNA mimic/inhibitor or expression vector transfection, TurboFect™ (Thermo, USA) was used. In accordance with the instructions, first, the interference sequence or miRNA mimic/inhibitor (5 µL) was mixed with serum-free medium (400 µL), then transfection reagent (6 µL) was added, and the mixture was mixed gently and allowed to stand for 15 min. Finally, the mixture was added to a six-well plate containing 600 µL/well complete medium. The human NR6A1 siRNA interference sequence (sense: 5’-GAG CAA CCA UGG UGA UAG UTT-3’, antisense: 5’-ACU AUC ACC AUG GUU GCU CTT-3’) and negative control siRNA sequence (sense: 5’-UUC UCC GAA CGU GUC ACG UTT-3’, antisense: 5’-ACG UGA CAC GUU CGG GAA ATT-3’) were purchased from GenePharma (Suzhou, China).

For the rescue experiment, after 36 h of transfection, the cells were treated with the mTOR phosphorylation activator MHY1485 (10 µM, Selleck, USA) for 12 h. Then, cell metabolism and proliferation were analyzed.

### Lentivirus Preparation

The lentiviral vector pLv[Exp]-EGFP: T2A: Puro-EF1A > NR6A1 containing the human NR6A1-coding sequence and the corresponding blank control vector were purchased from Guangzhou Trauer Biotechnology Co. Ltd. Three plasmids, the Gag-pol, Rev, and VSV-G plasmids (Guangzhou Trauer Biotechnology), were used for packaging lentiviral vectors. The cells were collected after transient transfection of the lentiviral vector into HEK293T cells for 48–72 h via TurboFectTM in vitro Transfection Reagent (Fermentas). The resulting supernatants were concentrated and filtered at 4 °C. The viral titer was determined to be 108 TU/ml by counting EGFP-positive cells via flow cytometry.

### Clinical specimens and ethics statement

Tissue microarray chips were obtained from Shanghai Qutdo Bitech Company, and each chip comprised 30 lung cancer tissue dots and 30 corresponding adjacent nontumor tissue dots. The study protocol was approved by the Shanghai Qutdo Biotech Company Ethics Committee. All experiments were performed in compliance with the relevant regulations, and all patients provided written informed consent.

**Fig. 1:**
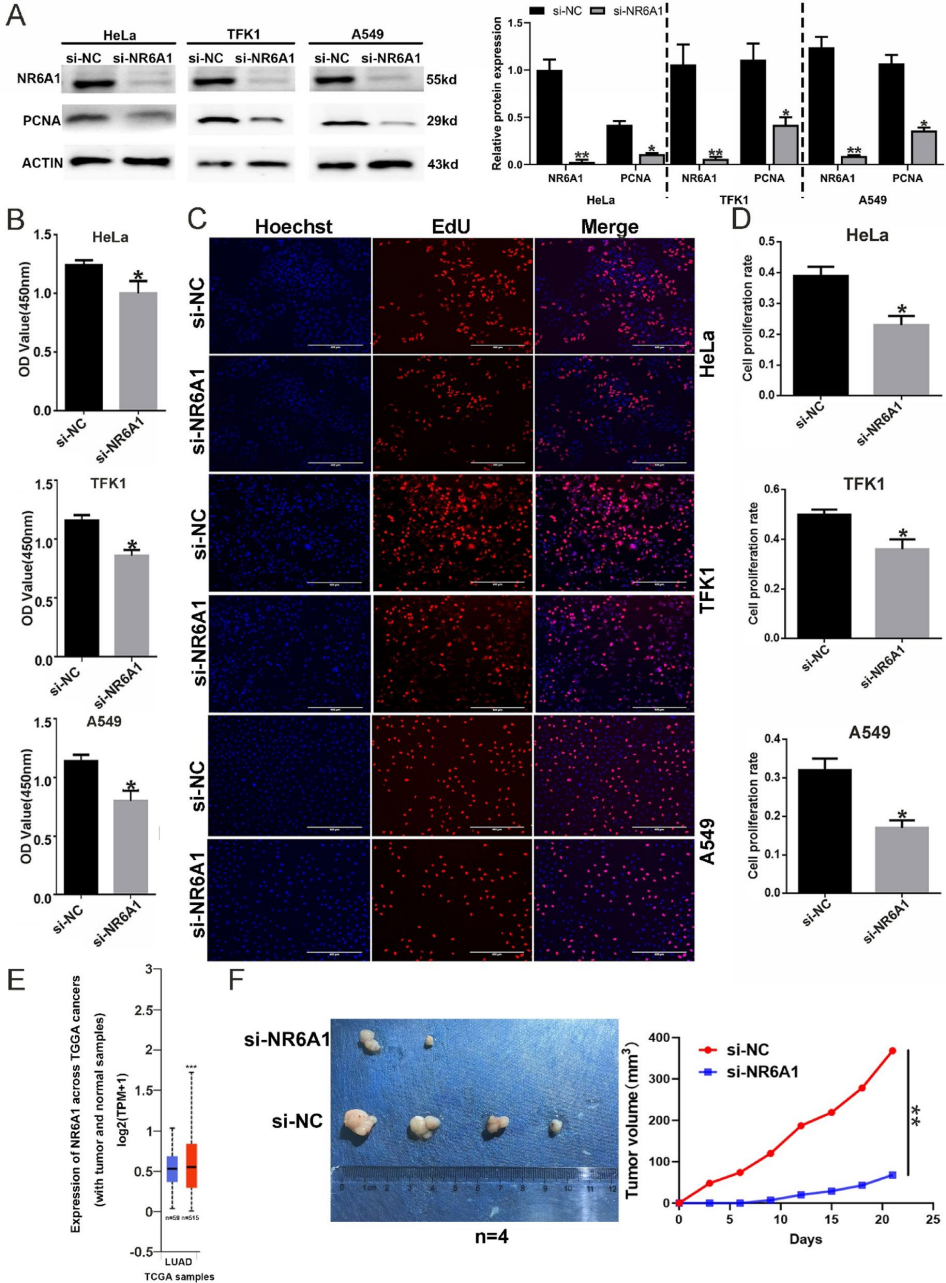
RNAi-mediated silencing of NR6A1 inhibits tumor cell proliferation. **A** The expression of NR6A1 and PCNA in tumor cells was detected by Western blotting after NR6A1 interference for 48 h. **B** CCK8 analysis was used to detect the proliferation of tumor cells after NR6A1 interference for 48 h. * indicates *P* < 0.05 compared with si-NC. (**C–D**) EdU staining was used to detect the proliferation rate of tumor cells after NR6A1 interference for 48 h (scale bar: 400 μm). * indicates *P* < 0.05 compared with si-NC. **E** The expression profile of NR6A1 in the Cancer Genome Atlas (TCGA) public database from the UALCAN website of lung adenocarcinoma compared with normal lung samples. **F** Interference with NR6A1 significantly suppressed the subcutaneous tumorigenic capacity of A549 cells in nude mouse xenograft models. * indicates *P* < 0.05 compared with si-NC.All quantitative data are presented as the means ± s.e.m.s

### Glucose uptake determination

The cells were seeded in a six-well plate (2 × 10^5^/well). The medium containing the supernatant was recovered 48 h after transfection. After centrifugation, the remaining glucose content in the fresh medium and supernatant was measured via a glucose assay kit (Nanjing Jiancheng, China) to analyze glucose uptake. For HeLa and TFK1 cells cultured in RPMI-1640 medium.A549 cells were maintained in DMEM medium.

### Lactic acid measurement

The cells were seeded in a six-well plate (2 × 10^5^/well). The supernatant medium was recovered 48 h after transfection, and the amount of lactic acid released from the supernatant was measured via a lactic acid assay kit (Nanjing Jiancheng, China) after centrifugation.

### ATP measurement

The cells were seeded in a six-well plate (2 × 10^5^/well). After transfection for 48 h, the cells were lysed with an ATP assay kit (Beyotime, China), centrifuged to obtain the supernatant, and mixed with diluted ATP test solution at a ratio of 1:1. The relative light unit (RLU) value was determined with a luminometer (Promega-GloMax, USA).

### Membrane potential detection

The cells were seeded in a six-well plate (2 × 10^5^/well). In accordance with the instructions of the Mitochondrial Membrane Potential Detection Kit (Beyotime, China), the cells were incubated with JC-1 for 48 h after transfection at 37 °C for 30 min and then washed and observed under a microscope (EVOS fl., USA).

###  Cell counting Kit-8 (CCK8) assay

After transfection for 48 h, the cells were seeded into 96-well plates at an initial density of 3 × 10^3^ cells per well, and five replicate wells were set for each group. After 24 h, CCK8 (10 µL/well; Beyotime, China) was added to the cells. After one hour of incubation at 37 °C, the absorbance was measured at 450 nm on a microplate reader (Thermo, USA).

### 5-Ethynyl-20-deoxyuridine (EdU) staining analysis

A BeyoClick™ EdU Cell Proliferation Kit with Alexa Fluor 488 was purchased from Beyotime. After siRNA transfection for 48 h, EdU was added to a six-well plate, which was subsequently incubated at 37 °C for 2 h. After permeation and BeyoClick reaction, the cells were stained with Hoechst 33,342 and observed under an eyepiece microscope (EVOS fl., USA).

### Western blot analysis

The following is the information concerning the antibodies used. NR6A1 (1:1000, Proteintech, China), PCNA (1:1000, CUSABIO, China), HK1 (1:1000, CUSABIO, China), HK2 (1:1000, CUSABIO, China), LDHA (1:1000, Proteintech, China), TIGAR (1:1000, CUSABIO, China), MFN1 (1:750, Solarbio, China), DRP1/2 (1:700, Wanleibio, China), APMKa1/2 (1:500, Wanleibio, China), p38 (1:1000, Proteintech, China), p-P38 (1:300, Wanleibio, China), ERK1/2 (1:1000, Proteintech, China), p-ERK1/2 (1:2000, CST, USA), mTOR (1:1000, Proteintech, China), p-mTOR (1:2000, CST, USA), and β-actin (1:5000, Bioworld, China). The dilution of the primary antibody was performed according to the instructions. After electrophoresis and membrane transfer were completed, the membrane was blocked with 5% skim milk at room temperature for one hour and incubated with the primary antibody at 4 °C overnight. The blot was washed and incubated with secondary antibody (Beyotime, China**)** for one hour and then detected with a chemiluminescent substrate (Beyotime, China) and an imaging system (Tanon, China). An anti-β-actin antibody was used as an internal control.

**Fig. 2:**
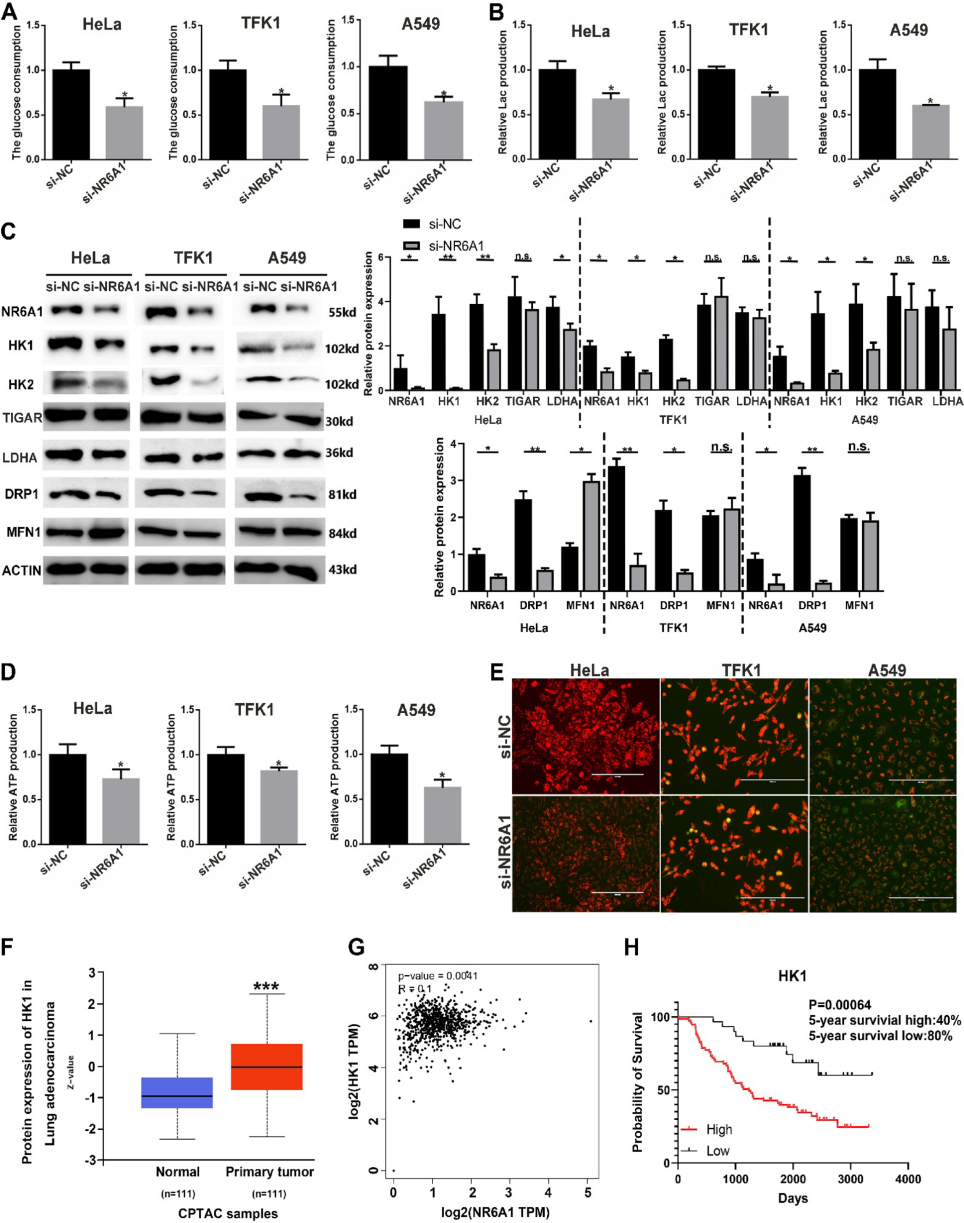
RNAi-mediated silencing of NR6A1 inhibits glycolysis in tumor cells. Glucose uptake.**A** Glucose uptake was detected in tumor cells after 48 h of NR6A1 interference. **B** Lactic acid production was detected in tumor cells after 48 h of NR6A1 interference. **C** Glycolytic enzyme proteins, DRP1 and MFN1 protein expression in tumor cells was detected via Western blotting after 48 h of NR6A1 interference. **D** ATP production was detected in tumor cells after 48 h of NR6A1 interference. * indicates *P* < 0.05 compared with si-NC.** E** JC-1 staining was used to detect the mitochondrial membrane potential of three cell lines after 48 h of NR6A1 interference (scale bar: 200 μm). **F** Expression of HK1 in primary tumor of lung adenocarcinoma compared with normal Lung samples detected in CPTAC samples.*** indicates *P* < 0.001 compared with normal samples.**G** Correlation analysis of NR6A1 and HK1 expression in lung adenocarcinoma, and the database from the HPA website.(H)5-year survival rate of different HK1 expression level in lung adenocarcinoma.All quantitative data are presented as the means ± s.e.m.s

### Dual-luciferase reporter assay

The fragments of HK1-3’-UTR-wild-type (WT) with miR-302a binding sites and its mutant (MUT) were digested with the restriction enzymes Xho I and Not I and then cloned and inserted into pmiR-RB-REPORT plasmids (Guangzhou RiboBio). The recombinant plasmids and miR-302a mimics/NC mimics were subsequently cotransfected into HEK293T cells via TurboFect™ (Thermo, USA). Relative Renilla luciferase activity was measured 48 h after transfection, and firefly luciferase activity was normalized to luciferase activity.

### Immunohistochemistry (IHC) and correlation analysis

Paraffin tissue array sections of lung adenocarcinoma (HLugA060PG02), including 30 tumor tissues and 30 adjacent noncancerous tissues, were purchased from Shanghai Outdo Biotech Co., Ltd. IHC was performed according to standard procedures. The primary antibodies anti-NR6A1 (1:100, ZenBioScience, China, 220732), anti-HK1 (1:100, Beyotime, China, AF1726), anti-p-mTOR (1:200, Bioss, bs-3494R) and DAB (ZSGB-Bio, China) were used for staining. The results were obtained with a digital slice scanner (3DHISTECH, Hungary). The IHC staining score was estimated according to the methods of our previous study (17). The IHC score was estimated as negative (0), weak (1), moderate (2), or strong (3). The extent of staining, defined as the percentage of positively stained cells, was scored as 1 (≤ 10%), 2 (11%−50%), 3 (51%−80%), or 4 (> 80%). The total immune reactive score (IRS) was obtained by multiplying the score of intensity and that of extent, ranking from negative (−) to > 6 (+++). The relevance of p-mTOR/NR6A1 and HK1/NR6A1 in lung adenocarcinoma patients was evaluated via Pearson correlation analysis.

### * In–vivo *xenograft experiment

The animal studies were approved by the Experimental Animal Ethics Committee of College of Biology, Hunan University. Six-week-old female nude mice (BALB/C) were injected subcutaneously with stable transfected 1 × 10^6^ A549 cells infected by si-NR6A1 or empty vector lentivirus, and 4 mice in each group. Tumor formation was investigated every 3 days, the tumor volume was calculated using the formula V = a × b^2^/2, where “a” is the long axis and “b” is the short axis. During the monitoring period, the changes in tumor volume were recorded to evaluate the tumor formation efficiency for 21 days.

### Statistical analysis

The data are presented as the means ± SDs of at least three separate experiments. Statistical differences between two groups were determined by Student’s t test, and one-way ANOVA was used when more than two groups were compared. *P* < 0.05 was considered to indicate a statistically significant difference. The data were analyzed with GraphPad Prism 8.0.1 (GraphPad Software Inc., San Diego, CA, USA).

##  Results

### RNAi-mediated Silencing of NR6A1 inhibits tumor cell proliferation

The expression profile of NR6A1 in the Cancer Genome Atlas (TCGA) public database from the UALCAN website (http://ualcan.path.uab.edu/cgi-bin/Pan-cancer.pl?genenam=NR6A1)indicated that NR6A1 is overexpressed in various cancers, suggesting that NR6A1 is related to tumorigenesis. Here, the cervical cancer cell line HeLa, the lung adenocarcinoma cell line A549, and the intrahepatic bile duct cancer cell line TFK1 were selected to investigate the effects of NR6A1 on tumor cell proliferation. Western blot analysis revealed that after NR6A1 was successfully silenced, the level of the proliferating cell nuclear antigen PCNA was significantly decreased (Fig. 1A). CCK8 analysis indicated that NR6A1 siRNA suppressed the proliferative viability of the three tumor cell lines (Fig. 1B). The results of EdU staining also revealed that NR6A1 siRNA reduced the proliferation rate of the three tumor cell lines (Fig. 1C, D). These results demonstrated that the RNAi-mediated silencing of NR6A1 inhibits tumor cell proliferation and that NR6A1 might play an oncogenic role in these tumors. Figure 1E shown that the expression of NR6A1 was significantly increased in lung adenocarcinoma compared with normal lung samples. Interference with NR6A1 significantly suppressed the subcutaneous tumorigenic capacity of A549 cells in nude mouse xenograft models(Fig. 1F).

###  RNAi-mediated Silencing of NR6A1 inhibits Glycolysis in tumor cells

Rapidly growing cells, such as cancer cells, primarily metabolize glucose via aerobic glycolysis. We speculate that the promoting effect of NR6A1 on proliferation may function by regulating glycolysis. Alterations in metabolism were detected in three tumor cell lines after interference with NR6A1, and the glucose uptake and lactate production of the cells were significantly reduced (Fig. 2A and B). The expression of the glycolytic rate-limiting enzymes hexokinase HK1 and HK2 was also dramatically downregulated.Furthermore, Western blot data indicated that the mitochondrial division protein DRP1 was significantly downregulated in all three cell lines, whereas the mitochondrial fusion protein MFN1 was upregulated only in HeLa cells(Fig. 2C). Changes in glucose metabolism in cancer cells are often accompanied by abnormalities in mitochondrial function. We investigated whether NR6A1 interference significantly reduced ATP production in the three tumor cell lines (Fig. 2D). Moreover, in HeLa and A549 cells, mitochondrial activity was significantly weakened, and the membrane potential was reduced, along with the occurrence of apoptosis (Fig. 2E).The normal division and fusion of mitochondria, which rely on the energy and material sources provided by glucose metabolism, are the basis of the mitosis of cells, ensuring normal cell proliferation. Our data showed that interference with NR6A1 can cause abnormal mitochondrial division and may decrease mitochondrial activity, which in turn inhibits glycolysis and cell proliferation. Figure 2F shown that the expression of HK1 was significantly increased in primary tumor of lung adenocarcinoma compared with normal Lung samples.Furthermore, NR6A1 expression exhibits a positive correlation with HK1 expression in lung adenocarcinoma (*R* = 0.1, *P* = 0.0041)(Fig. 2G).Patients with high HK1 expression exhibited a significantly worse 5-year survival rate (40%) compared to those with low HK1 expression (80%) in lung adenocarcinoma, demonstrating an oncogenic role for HK1 in lung adenocarcinoma(*P* = 0.00064))(Fig. 2H).

### Activation of mTOR phosphorylation can restore the proliferation and glycolytic phenotype of lung cancer cells with NR6A1 knockdown

**Fig. 3:**
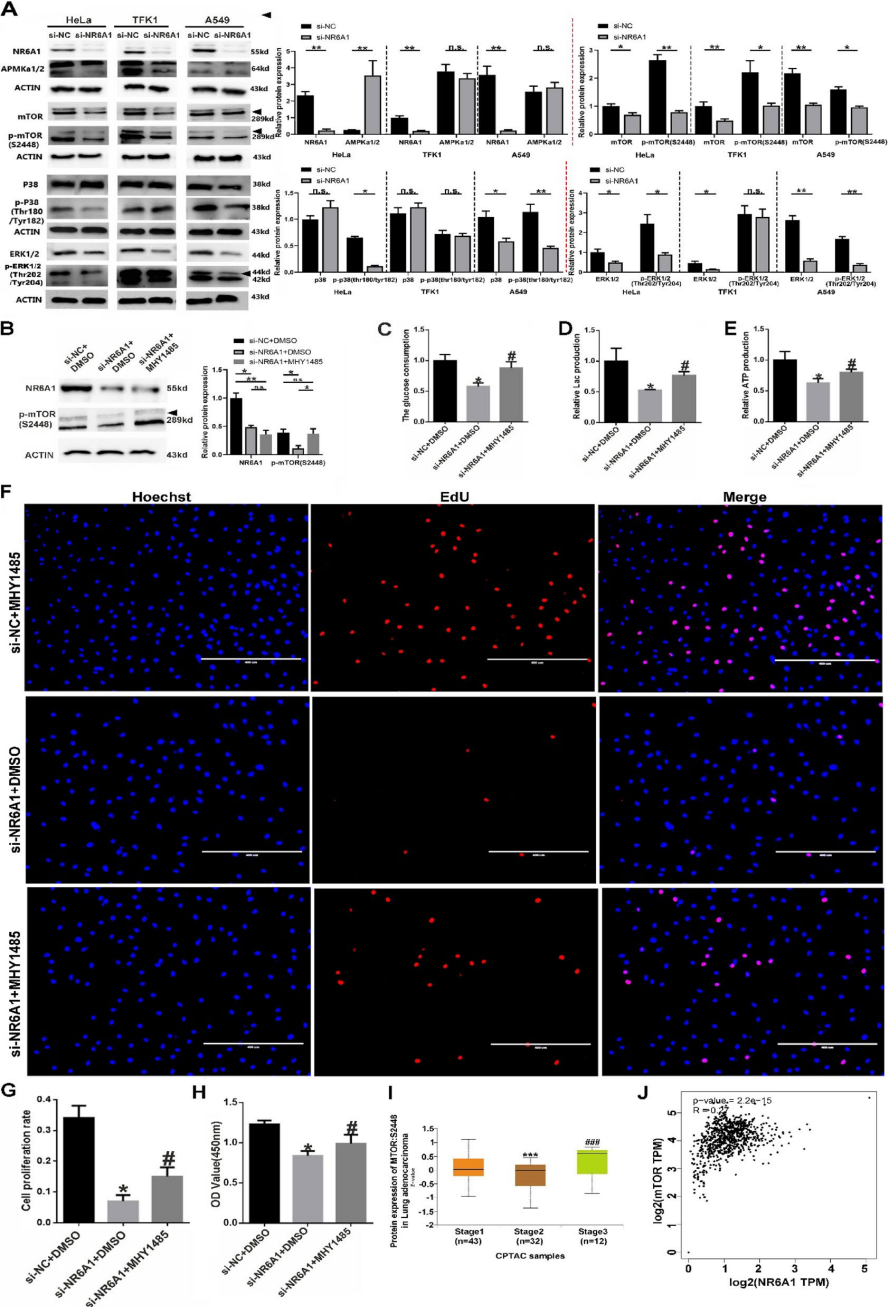
Activation of mTOR phosphorylation restores the proliferation and glycolytic phenotype in A549 cells with NR6A1 interference. **A** Total protein levels and serine phosphorylation levels of AMPK, mTOR, p38 and ERK were detected in HeLa, A549 and TFK1 cells by Western blotting after 48 h of NR6A1 interference. **B** The protein expression of NR6A1 and p-mTOR in MHY1485-treated A549 cells with NR6A1 interference was detected via Western blotting. (**C**) Glucose uptake was analyzed in MHY1485-treated A549 cells with NR6A1 interference. **D** Lactic acid production was analyzed in MHY1485-treated A549 cells with NR6A1 interference. **E** ATP production was analyzed in MHY1485-treated A549 cells with NR6A1 interference. * indicates *P* < 0.05 compared with si-NC + DMSO, and # indicates *P* < 0.05 compared with si-NR6A1 + DMSO. ** F** EdU staining was used to detect the cell proliferation rate in MHY1485-treated A549 cells with NR6A1 interference (scale bar: 400 μm). **G** Quantification of the EdU results. * indicates *P* < 0.05 compared with si-NC + DMSO, and # indicates *P* < 0.05 compared with si-NR6A1 + DMSO. All the quantitative data are presented as the means ± s.e.m.s ** H** Cell proliferation was detected via the CCK8 method in MHY1485-treated A549 cells with NR6A1 interference; * indicates *P* < 0.05 compared with the si-NC + DMSO group, and # indicates *P* < 0.05 compared with the si-NR6A1 + DMSO group. ** I** Expression of p-mTOR(Ser2448) in different stage of lung adenocarcinoma samples detected in CPTAC samples.*** indicates *P* < 0.001 compared with stage1 samples;###indicates *P* < 0.001 compared with stage2 samples.**J** The correlation between NR6A1 and mTOR mRNA levels in patients with lung adenocarcinoma was estimated via Pearson correlation analysis of data from the TCGA database.All quantitative data are presented as the means ± s.e.m.s

To determine whether and which cellular signal is involved in NR6A1-mediated regulation of glycolysis and cell proliferation, we examined several key signaling pathways, including AMP-activated protein kinase (AMPK), mammalian target of rapamycin (mTOR), extracellular signal-regulated kinase (ERK) and p38 kinase. Western blot analysis (Fig. 3A) revealed that, after interference with NR6A1 in the three tumor cell lines, AMPK was the least affected, and the total protein levels and serine phosphorylation (Ser2448) of mTOR were significantly decreased. However, in both HeLa cells and A549 cells, only the phosphorylation levels of ERK (Thr202/Tyr204) and p38 (Thr180/Tyr182) were decreased. Therefore, we speculate that mTOR signaling may be the most important mechanism by which NR6A1 regulates tumor cell glycolysis and proliferation.

In view of the similar biological effects of NR6A1 on these three tumor cell lines, we used A549 lung cancer cells as the experimental materials in the following study. MHY1485, an mTOR phosphorylation activator, was added to NR6A1-siRNA-treated A549 cells to analyze whether NR6A1 functions in glycolysis and proliferation through mTOR signaling. The western blot results revealed that the level of phosphorylated mTOR in A549 cells was significantly increased after MHY1485 treatment (Fig. 3B), accompanied by increases in glucose uptake and lactic acid and ATP production (Fig. 3C, D and E). EdU staining (Fig. 3F and G) and CCK8 analysis (Fig. 3H) revealed that in NR6A1-siRNA-treated cells, the addition of MHY1485 restored the proliferative phenotype of A549 cells to a certain extent, suggesting that NR6A1 promotes tumor cell glycolysis and proliferation via mTOR signaling. Analysis of CPTAC data revealed significantly increased protein expression of p-mTOR (Ser2448) in stage III lung adenocarcinoma patients(Fig. 3I).In addition, Pearson correlation analysis of data from the TCGA database revealed that the levels of the mTOR and NR6A1 mRNAs were positively correlated in patients with lung adenocarcinoma (*R* = 0.27, *P* = 2.2e-15) (Fig. 3J).

### Correlations among NR6A1/p-mTOR and NR6A1/HK1 in clinical lung adenocarcinoma tissue samples

**Fig. 4:**
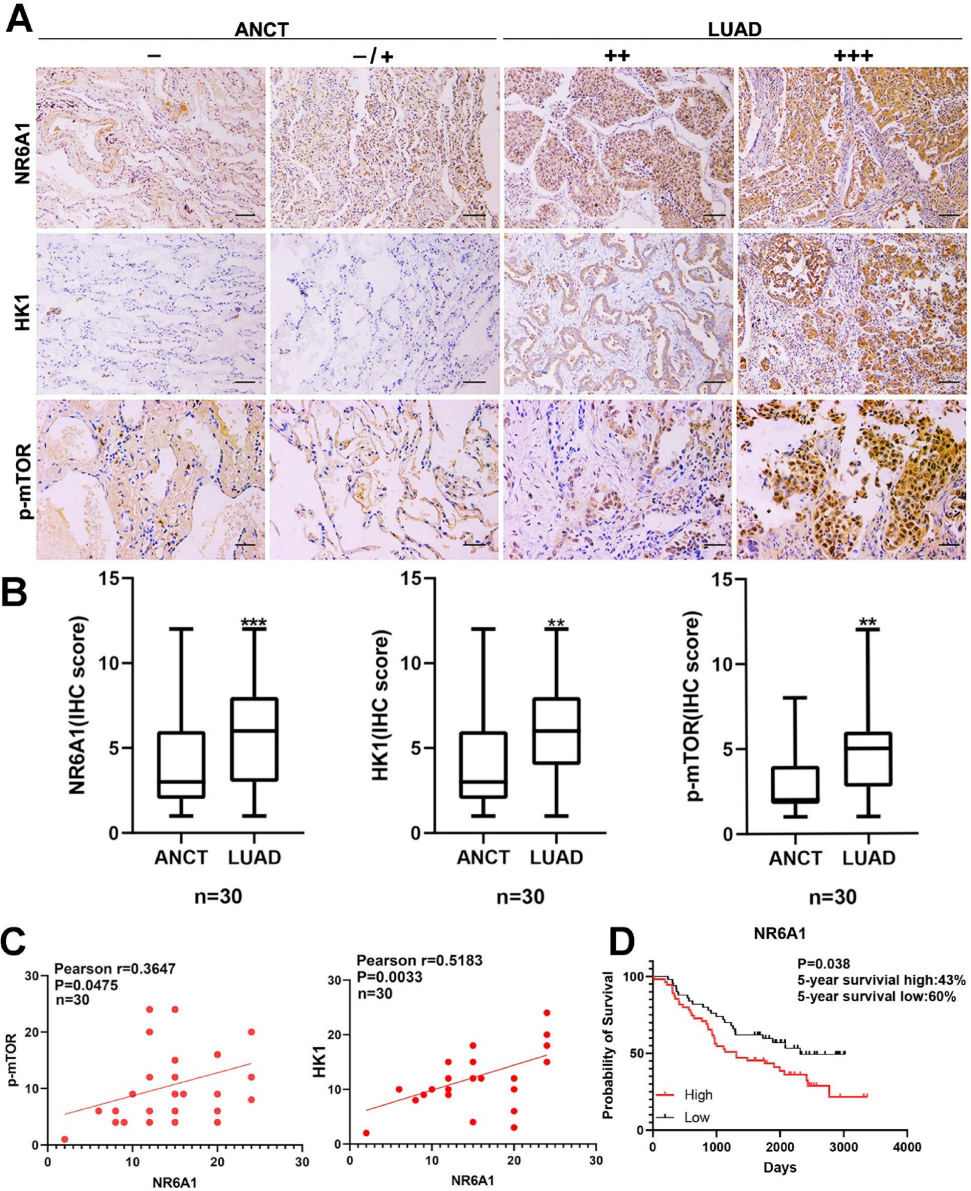
Correlation of NR6A1/HK1 and NR6A1/p-mTOR in clinical LUAD tissue samples. **A** Immunohistochemistry analysis of NR6A1, HK1, NR6A1 and p-mTOR expression in both ACNT and LUAD tissues (scale: ×200). ACNT, adjacent noncancerous tissue; LUAD, lung adenocarcinoma. **B** Immunohistochemistry scores of NR6A1, HK1, and p-mTOR in both ACNT and LUAD tissues. **P < 0.01, ***P < 0.001. **C** The correlations between NR6A1 and HK1 (*r* = 0.5183, *P* = 0.0033), NR6A1 and p-mTOR (*r* = 0.3647, *P* = 0.0475) expression in LUAD tissues were analyzed via immunohistochemical staining. The correlation was deemed significant and positive when *P* < 0.05. The IHC score was estimated as negative (0), weak (1), moderate (2), or strong (3). The extent of staining, defined as the percentage of positively stained cells, was scored as 1 (≤ 10%), 2 (11%−50%), 3 (51%−80%), or 4 (> 80%). The total immune reactive score (IRS) was obtained by multiplying the score of intensity and that of extent, ranking from negative (−) to > 6 (+++).(D)5-year survival rate of different nr6a1 expression level in lung adenocarcinoma. All quantitative data are presented as the means ± s.e.m.s

Since NR6A1 may play an oncogenic role in tumors through the p-mTOR and miR-302a/HK1 pathways, we next examined the correlations between NR6A1 and p-mTOR, NR6A1 and HK1 in clinical LUAD tissue samples via IHC analysis with a LUAD tissue array. Compared with those in the adjacent noncancerous tissue (ANCT) group, high expression of NR6A1 was observed in the lung adenocarcinoma (LUAD) group, whereas the protein levels of HK1 and p-mTOR were high in the LUAD group (Fig. 4A, B). Pearson correlation analysis based on the IHC score (Fig. 6C) revealed that NR6A1 and HK1 were positively correlated (*R* = 0.6028, *P* = 0.0004), as were NR6A1 and p-mTOR (*R* = 0.4301, *P* = 0.0177).Patients with high NR6A1 expression exhibited a significantly worse 5-year survival rate (43%) compared to those with low HK1 expression (60%) in lung adenocarcinoma, demonstrating an oncogenic role for nr6a1 in lung adenocarcinoma(*P* = 0.038)(Fig. 4D).

**Fig. 5:**
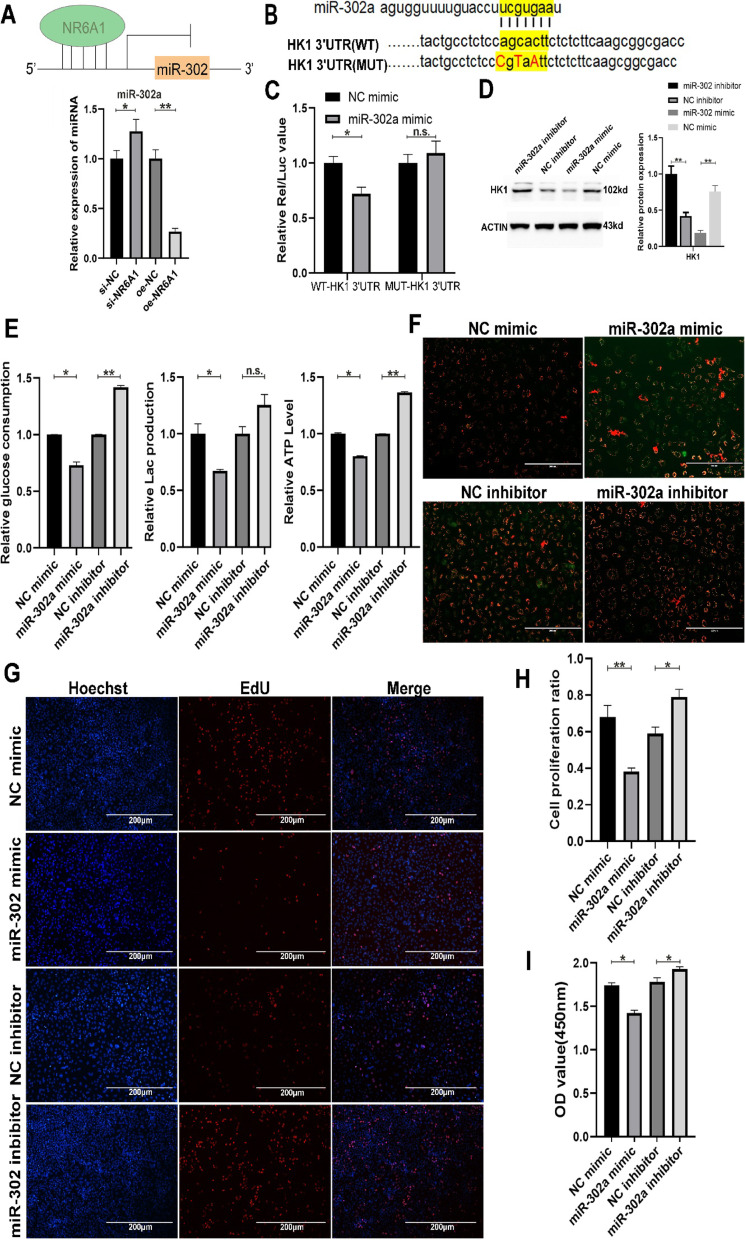
miR-302a/HK1 inhibits glucose metabolism and cell proliferation in A549 cells. **A** A qPCR assay was used to detect the miR-302a level after 48 h of NR6A1 interference or overexpression in A549 cells. **B** The binding site of miR-302a on the HK1 mRNA 3’UTR sequence was analyzed, and the highlight with yellow represents the miR-302a binding site. **C** Dual-luciferase reporter gene experiments were performed to detect the regulatory relationship between miR-302a and HK1. **D** Western blotting was used to detect the protein level of HK1 after transfection with the miR-302a mimic or inhibitor. **E** The relative glucose consumption, relative lactic acid production and relative ATP level were detected in A549 cells transfected with the miR-302a mimic or inhibitor. **F** Mitochondrial membrane potential was analyzed after transfection with the miR-302a mimic or inhibitor (scale bar: 200 μm). EdU (**G–H**) and CCK8 (** I**) assays were performed to detect the proliferation of A549 cells after transfection with the miR-302a mimic or inhibitor. * indicates *P* < 0.05, ** indicates *P* < 0.01 and n.s. indicates *P* > 0.05. All quantitative data are presented as the means ± s.e.m.s

###  NR6A1 promotes glucose metabolism and proliferation in lung cancer cells through the miR-302a/HK1 axis

NR6A1 was recognized as a transcriptional inhibitor in previous studies [35–37]. However, our present study revealed that NR6A1 may promoted the expression of HK1/2, implying that there might be an indirect regulatory relationship between them. Wang et al. identified the noncoding RNA miR-302a as the target gene of NR6A1 in embryonic stem cells [38] and reported that NR6A1 directly binds to the DR0 site on the promoter of the miR-302a precursor gene, thereby inhibiting the transcription of miR-302a. We wondered whether NR6A1 downregulates miR-302a in lung cancer cells. After NR6A1 was silenced or overexpressed in A549 cells, quantitative RT‒PCR revealed that interference with NR6A1 increased the expression of miR-302a, whereas overexpression of NR6A1 decreased the level of miR-302a, indicating that NR6A1 exerted transcriptional inhibition ontranscriptionally inhibited miR-302a in lung cancer cells (Fig. 5A). Interestingly, the 3’UTR of the HK1 gene was predicted to contain the miR-302a binding site (Fig. 5B). Then, we constructed recombinant dual-luciferase reporter gene vectors containing wild-type (WT) and mutant (MUT) HK1 3’UTR,3’UTRs and cotransfected them with a miR-302a mimic/NC mimic into HEK293T cells. The results of the reporter gene assay revealed that miR-302a significantly inhibited the activity of HK1 3’UTR-WT but had no significant effect on HK1 3’UTR-MUT (Fig. 5C). The western blot results further confirmed that the overexpression of miR-302a inhibited the expression of HK1; conversely, the miR-302a inhibitor promoted HK1 expression (Fig. 5D), indicating that HK1 is the target gene of miR-302a in lung cancer cells. We subsequently detected the effect of miR-302a on metabolism in A549 cells and found that miR-302a inhibited glucose consumption and lactic acid and ATP production (Fig. 5E) and inhibited mitochondrial function (Fig. 5F). Furthermore, the results of the EdU (Fig. 5G, H) and CCK8 (Fig. 5I) assays revealed that miR-302a inhibited the proliferation of lung cancer cells. The above data indicate that miR-302a inhibits glucose metabolism, mitochondrial function and cell proliferation and that the miR-302a/HK1 axis may contribute to the effect of NR6A1 on reprogramming glycolysis in lung cancer cells.

**Fig. 6:**
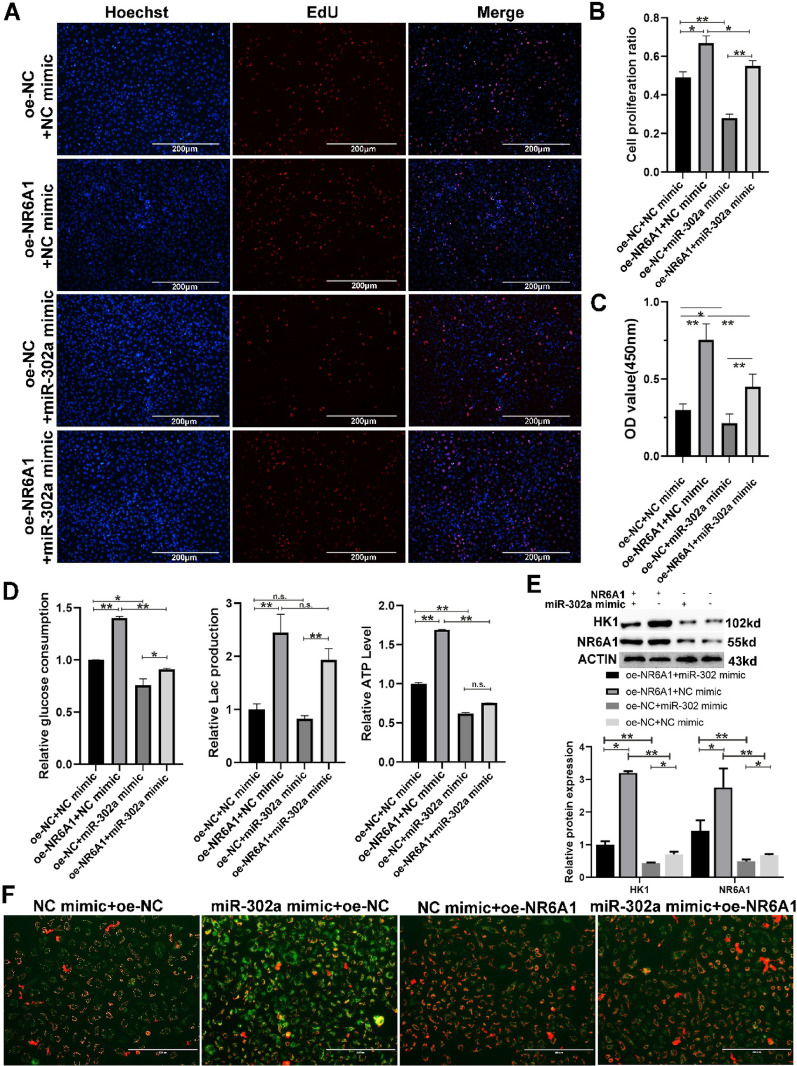
miR-302a can restore glycolysis and the proliferative phenotype in A549 cells overexpressing NR6A1. (**A–B**) EdU staining and** C** the CCK8 assay were used to detect the proliferation ability of A549 cells after coinfection with miR-302a and NR6A1 for 48 h. **D** The relative glucose consumption, relative lactic acid production and relative ATP level were analyzed in A549 cells coinfected with miR-302a and NR6A1. **E** Western blotting was used to detect the HK1 protein in A549 cells coinfected with miR-302a and NR6A1. **F** Mitochondrial function was detected in A549 cells coinfected with miR-302a and NR6A1 (scale bar: 200 μm). * indicates *P* < 0.05, ** indicates *P* < 0.01 and n.s. indicates *P* > 0.05. All quantitative data are presented as the means ± s.e.m.s

To determine whether NR6A1 reprograms glucose metabolism through the miR-302a/HK1 pathway, we performed a series of functional rescue experiments. EdU (Fig. 6A, B) and CCK8 (Fig. 6C) assays revealed that cotransfection of the NR6A1 overexpression vector and miR-302a mimics inhibited the proliferation of A549 cells compared with that of the NR6A1 overexpression group and promoted the proliferation of cells compared with that of the miR-302a mimics group. The analysis of metabolic levels revealed that glucose consumption and ATP production were significantly lower in the NR6A1 and miR-302a cotransfection group than in the NR6A1 overexpression group. Although there was no significant difference in lactic acid production, the cotransfection group presented a reduction in lactic acid compared with the NR6A1-overexpressing group (Fig. 6D). Compared with NR6A1 overexpression, cotransfection of NR6A1 and miR-302a significantly suppressed HK1 protein expression (Fig. 6E), indicating that NR6A1 indeed upregulated HK1 expression by directly inhibiting miR-302a. In addition, mitochondrial function analysis via rescue experiments confirmed the inhibitory relationship between NR6A1 and miR-302a (Fig. 6F). Taken together, the results of rescue experiments revealed that NR6A1 promotes glucose metabolism and proliferation through miR-302a/HK1 in lung cancer cells.

## Discussion

Metabolic reprogramming of cancer cells is usually mediated by oncogenic signals [39]. Our data revealed that NR6A1 plays an oncogenic role in several types of cancer cells, such as cervical cancer, lung adenocarcinoma and intrahepatic bile duct cancer cells. Knockdown of NR6A1 leads to decreases in glucose uptake, lactic acid production and ATP levels, as well as the downregulation of mTOR at the protein and phosphorylation levels.

Nuclear receptors extensively participate in metabolic processes. As transcription factors, nuclear receptors such as PPARs and Nur77 directly regulate the expression of key enzymes and pathways involved in glucose metabolism [40, 41].PPARγ modulates genes associated with glycolysis, gluconeogenesis, and insulin sensitivity by recruiting coactivators or corepressors, thereby influencing cellular glucose uptake and utilization.Nur77 deficiency can lead to dysregulation of glucose metabolism, manifested as hyperglycemia, reduced insulin secretion, and β-cell dysfunction. This occurs mechanistically through downregulation of insulin gene expression and dysregulation of glycolysis/gluconeogenesis pathways.Cancer cells exhibit metabolic reliance on glycolysis for energy production (the Warburg effect), even under aerobic conditions. Aberrant inactivation of the nuclear receptor PPARγ may contribute to the upregulation of key glycolytic enzymes such as Hexokinase 2 (HK2) and Lactate Dehydrogenase A (LDHA), enhancing tumor cell proliferative capacity.Prior to this study, the nuclear receptor NR6A1 had not been investigated in the context of glucose metabolism. Our research provides preliminary evidence demonstrating a functional role for NR6A1 in tumor glucose metabolism.In the process of glycolysis, HKs are the first enzymes that can convert glucose to glucose 6-phosphate. In contrast to that in normal tissues, increased expression of HKs such as HK1 has been observed in many types of cancer, such as cervical cancer [42] and bile duct carcinoma [43]. Here, Our study demonstrates that NR6A1 promote the expression of HK1 indirectly. To determine the regulatory relationship between NR6A1 and HK1, we screened miRNAs targeting the HK1 gene and found that miR-302a is not only the target gene of NR6A1 but also has conserved recognition sites in the 3’-UTR sequence of HK1 mRNA, suggesting that miR-302a may act as a linker between NR6A1 and HK1. Through a series of experiments, including bioinformatics analysis, dual-luciferase reporter gene expression, RT‒qPCR and Western blot analysis, we proved that NR6A1 could inhibit miR-302a in lung cancer cells, thereby indirectly promoting the expression of HK1. The results of functional rescue experiments, such as glucose consumption detection, lactic acid production analysis, ATP production analysis, and EdU and CCK8 assays, further demonstrated that NR6A1 promotes glucose metabolism and HK1 expression by targeting the degradation of miR-302a, thus promoting the proliferation and survival of lung cancer cells.

Like most cancer/testis (CT) genes, NR6A1 is expressed mainly in male gonads, repressed in most healthy somatic tissues and derepressed in various somatic malignancies. CT genes are considered useful biomarkers and immunotherapy targets because of their tissue-specific expression pattern and immunogenicity [44, 45]. Among the different types of tumors, non-small cell lung carcinoma is grouped as having high CT gene expression [46]. Thus, in the present study, the role and mechanism of NR6A1 in glycolysis were investigated in lung adenocarcinoma, and the clinical association of NR6A1 with lung adenocarcinoma was analyzed. We confirmed that high expression of NR6A1 in lung adenocarcinoma tissues was significantly positively correlated with HK1 and p-mTOR expression. However, while integrating external database findings with our tissue microarray results (*n* = 30) has preliminarily revealed the expression profile of NR6A1 in lung adenocarcinoma and its correlation with HK1, the sample size remains constrained. Future validation should be conducted using expanded clinical cohorts incorporating normal pulmonary tissue samples.

Numerous studies have shown that mTOR is an essential molecule that regulates cell growth and senses the nutritional status of cells according to environmental and physiological cues [47]. The hyperfunction of mTOR, such as the phosphorylation of mTOR at Ser2448, is the key factor that leads to the Warburg effect in tumor cells. In the present study, through the introduction of the mTOR phosphorylation activator MHY1485 into NR6A1-siRNA-treated A549 cells, we observed that MHY1485 (10 µM) could restore the glycolytic and proliferative phenotypes of cells to a certain extent, suggesting that the role of NR6A1 in cell proliferation may relies on p-mTOR-induced glycolysis. A limitation of this study lies in elucidating how NR6A1, functioning as a transcriptional repressor, regulates the mTOR signaling pathway. However, it has been reported that siRNA-mediated knockdown of NR6A1 upregulates insulin receptor (INSR) expression and potentiates insulin-induced mTOR/AKT phosphorylation in HepG2 cells, partially through miR-205-5p [19]. Based on our findings, we propose that NR6A1 may orchestrate cellular bioenergetics metabolism across diverse tumor cell types via mTOR signaling. Subsequent studies employing integrated metabolic profiling (e.g., oxygen consumption rate detection assays) could comprehensively characterize the role of the NR6A1-mTOR axis in tumor cell metabolic reprogramming.

**Fig. 7:**
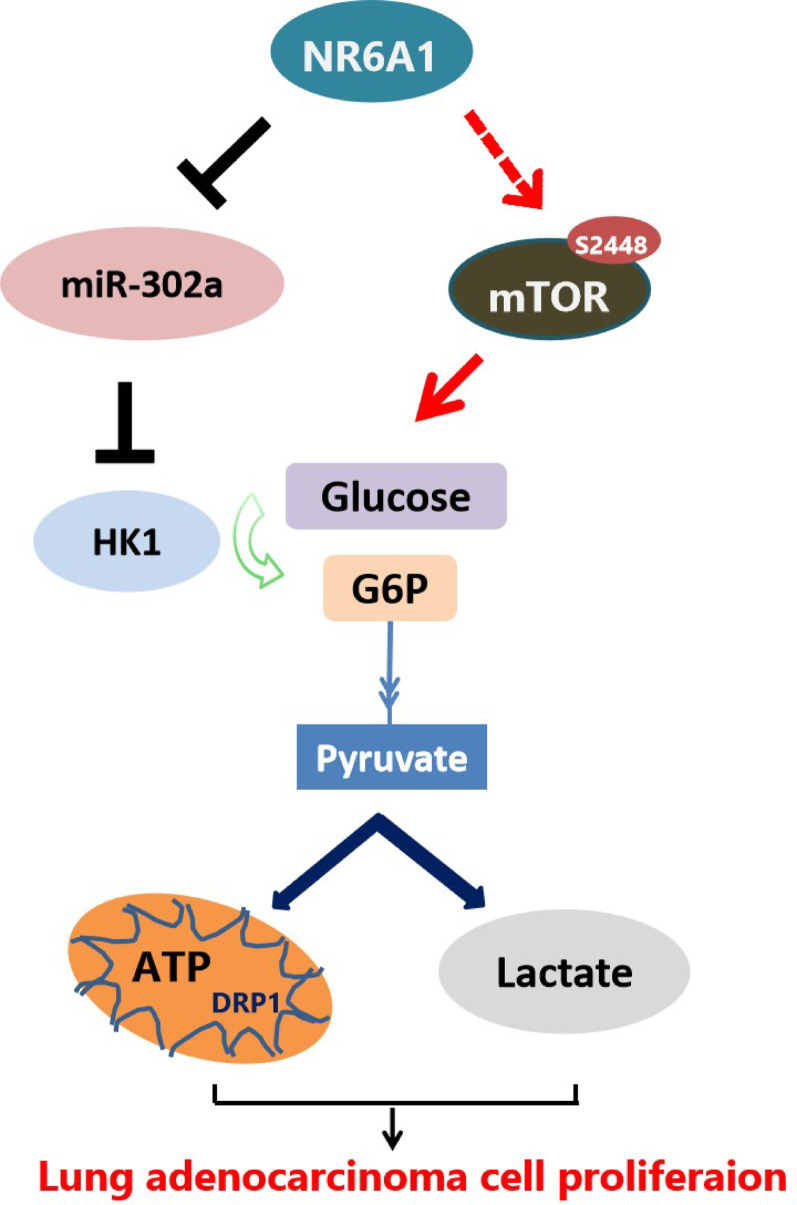
schematic model showing that NR6A1 promotes tumor cell proliferation by reprogramming glycolysis via the miR-302a/HK1 pathway, and may also influence the p-mTOR signaling

mTOR is one of the key mechanisms used by cancer cells to enhance their growth capacity, driving a wide range of biological processes to support the metabolic transformation of tumors. Abnormal activation of the mTOR pathway is associated with cell proliferation and survival. Ser2448 is a widely studied site, often considered a marker of mTORC1 complex activity. HK1 is overexpressed in various cancers and is associated with poor prognosis68, driving tumor progression by promoting glycolysis. This study found that NR6A1 can regulate both mTOR phosphorylation and HK1 expression. However, the hierarchical relationship and the relationship among NR6A1, mTOR, and HK1 need further clarification. It is highly plausible that NR6A1 modulates mTOR activity through altering tumor cell metabolic states, and mTOR may subsequently regulate cellular metabolism and proliferation. Taken together, the present research is the first to describe the important regulatory role of NR6A1 in lung adenocarcinoma glycolysis. NR6A1 activated glycolysis through p-mTOR signaling and the miR-302a/HK1 axis and ultimately promoted tumor cell proliferation(Fig. 7). In the future, it may be possible to combine NR6A1-targeted therapy with existing standard treatments, such as chemotherapy or immunotherapy, to synergistically enhance the anti-tumor effect.

## Data Availability

The datasets used and/or analyzed during the current study are available from the corresponding author upon reasonable request.

## Abbreviations

AMPK: AMP-activated protein kinase
ANCT: Adjacent noncancerous tissues
CT: Cancer/Testis
CCK8: Cell Counting Kit-8
ERK: Extracellular signal-regulated kinase
EdU: 5-Ethynyl-20-deoxyuridine
IHC: Immunohistochemistry
LUAD: Lung adenocarcinoma
mTOR: Mammalian target of rapamycin
MUT: Mutant
NR6A1: Nuclear receptor subfamily 6 group member 1
si-NC: siRNA-Negative Control
TCGA: The Cancer Genome Atlas
WT: Wild-type
